# The Role of Haemostasis Course in Increasing Knowledge and Skills in Managing Upper Gastrointestinal Bleed of the Delegates: A British Society of Gastroenterology’s Endoscopy Quality Improvement Programme, Yorkshire Project

**DOI:** 10.7759/cureus.15511

**Published:** 2021-06-08

**Authors:** Varshil Mehta, Simran Kang, Mo Thoufeeq

**Affiliations:** 1 Internal Medicine, Lister Hospital, Stevenage, GBR; 2 Surgery, Lister Hospital, Stevenage, GBR; 3 Gastroenterology, Sheffield Teaching Hospitals National Health Service (NHS) Foundation Trust, Sheffield, GBR

**Keywords:** quality improvement projects, gastroenterology and endoscopy, acute gastrointestinal bleed, haemostasis, bleeding

## Abstract

Introduction

An acute upper gastrointestinal bleed (AUGIB) is a fatal and prevalent medical emergency if not appropriately treated in a timely fashion.

Aim

The aim of this project was to compare the knowledge and skills of the participants in managing upper gastrointestinal bleeding (UGIB) before and after a one-day UGIB haemostasis course.

Methods

A one-day haemostasis course in line with the British Society of Gastroenterology’s Endoscopy Quality Improvement Project Initiative was organised at the Sheffield Teaching Hospitals National Health Service (NHS) Trust. The course included lectures on UGIB and its management, which was followed by hands-on training on adrenaline injection, variceal banding, clip placement, thermal therapy, Hemospray® use, Sengstaken-Blakemore tube placement, and Danis stent placement via porcine or plastic models. Pre- and post-course feedback questionnaires consisting of self-assessed ratings related to knowledge, skills, and behaviour relevant to UGIB were offered to all delegates. Two-tailed Wilcoxon signed-rank test was used to compare the results.

Results

A total of 36 individuals attended the course. Delegates had an average endoscopy procedure count of 583. The cohort ranged from different fields of medicine, including gastroenterology consultants and junior doctors. Ten of the delegates were Joint Advisory Group-certified in upper gastrointestinal endoscopy. Feedback datasheets were returned by 22 delegates. Significant improvements were reported post-course (p < 0.001), especially in the hands-on and behavioural areas.

Conclusion

Overall, there was a significant improvement in the knowledge, procedural skills, and confidence of the delegates in the management of an AUGIB post-course. We recommend not only to include this course in gastrointestinal training but also to conduct a course such as this for consultants and junior doctors who wish to undergo gastrointestinal training in the future.

## Introduction

Acute upper gastrointestinal bleeding (AUGIB) is currently one of the most common emergency presentations, with an estimated incidence of 134 per 100,000 in the population and a mortality rate of approximately 10% in the United Kingdom (UK) [1-2].

In order to manage AUGIB, multiple guidelines have been published by the Asia-Pacific Working Group Consensus, the National Institute for Health and Care Excellence, the Scottish Intercollegiate Guidelines Network in conjunction with the British Society of Gastroenterology and the European Society of Gastrointestinal Endoscopy [3-6]. In 2015, the difference in standards of practice in managing UK patients with AUGIB was noted by the National Confidential Enquiry into Patient Outcome and Death (NCEPOD). Concerns were raised regarding suboptimal upper gastrointestinal bleeding (UGIB) management [7].

Around 77% of the gastroenterology units across the UK have dedicated AUGIB emergency services. However, the delivery of its services varies with few providing outstanding services while some provide below-average services [8]. Previous studies have shown that these current hospital emergency services are inadequately resourced, especially out-of-hours, with few fully trained in endoscopic procedures [9-10]. The major reason is a lack of training of the gastroenterology registrars/fellows in managing AUGIB. Observations have highlighted that training in endoscopic management of UGIB may not be delivered in a standardised pattern in gastroenterology training programmes [11-12].

In addition to this, a few studies have also reported that the gastroenterology trainees have, in fact, been disadvantaged by a reduction in their procedural training due to the European Working Time Directive (EWTD) and the New Deal, resulting in a total loss of approximately 1,400 hours per year [13-17]. Hence, only 15% of the total endoscopies were performed by gastroenterology trainees in 2011 versus 76% performed by them in 1996 (p < 0.001). In fact, 23% (57/245) of the gastroenterology trainees felt that “they would not be competent in AUGIB endoscopy by completion of specialty training” because of the reduction in training time [14].

The present study, as a part of a quality improvement project, was conducted in order to observe if there is any improvement in the quality by conducting a UGIB haemostasis course for not only gastroenterology trainees but also other medical or para-medical members.

This study was also presented as an abstract at the European Society of Gastroenterology Endoscopy (ESGE) Days Conference in 2020, Dublin, Ireland [18].

## Materials and methods

A one-day course was arranged for doctors and nurses involved in managing AUGIB at the Sheffield Teaching Hospital. This course was in line with the British Society of Gastroenterology’s Endoscopy Quality Improvement Project (BSG EQIP) initiative and in conjunction with the Joint Advisory Group (JAG).

Lectures were given on pre-endoscopic consideration, risk assessment, potential aetiologies of UGIB, pre-endoscopy management and timing of endoscopy, management of variceal, non-variceal, and atypical UGIB, and endoscopy report writing (given to all attendees, including junior doctors and nursing staff). Subsequent hands-on training on adrenaline injection, clip placement, variceal banding, thermal therapy, use of haemostatic powder (Hemospray®, Cook Medical, Winston-Salem, NC), Sengstaken-Blakemore tube placement, and Danis stent placement was also arranged via porcine or plastic models to all specialty trainees in year 3 and above (registrars/fellows and consultants). Personalised training was given to each participant by competent consultants.

Feedback questionnaires consisting of self-assessed pre- and post-course ratings related to knowledge, skills, and behaviour relevant to UGIB were given to all delegates. Each delegate had to self-score each question from a maximum of 10 points (on a scale of 10 points with 10 denoting excellent knowledge and 0 denoting having no knowledge for the given subject). The questionnaire was divided into four sections: (1) knowledge of upper gastrointestinal (GI) bleed (pre-endoscopic consideration, risk stratification systems, potential pathologies responsible for UGIB, medical management of variceal and non-variceal GI bleed); (2) understanding or knowledge of the procedure for managing the UGIB (procedures included adrenaline infiltration, clip placement, variceal banding, thermal therapy, Hemospray use, Sengstaken-Blakemore tube placement, and Denis stent placement); (3) performing the procedure (procedures included adrenaline infiltration, clip placement, variceal banding, thermal therapy, Hemospray use, Sengstaken-Blakemore tube placement, and Denis stent placement); and (4) behaviour (confidence of instructing during the procedure).

Two-tailed Wilcoxon signed-rank test was used to compare the results, and p-value of 0.05 or lesser than that was considered statistically significant.

## Results

The course was attended by 36 individuals ranging from different areas of specialisation and seniority, with an average endoscopy procedure count of 583.

Delegates included 10 JAG-certified consultants in UGI endoscopy. Four were gastroenterology consultants and one was a surgical consultant. Eight delegates were gastroenterology trainees/registrar, one surgical registrar, one medical registrar, and three junior doctors, with the remainder being nurse practitioners. Junior doctors and nurse practitioners attended only the lectures regarding UGIB and procedures.

Feedback datasheets were collected. A total of 22 out of the 36 participants completed the feedback forms. There was a statistically significant improvement (p < 0.001) post-course in almost all areas. Notable areas of improvement were the hands-on and behavioural components (Table 1).

**Table 1:** Comparison Between Pre- and Post-Course Knowledge, Skills, and Behaviour p < 0.05 is statistically significant. Scores were out of 10. GI, gastrointestinal; JAG, Joint Advisory Group; ST3, specialty trainees (fellows); UGIB, upper gastrointestinal bleeding

Course objective	Median Score		p-Value (two-tailed Wilcoxon test)
	Pre-course	Post-course	
Knowledge			
Pre-endoscopic consideration	7	9	<0.001
Risk stratification systems	7	8	0.02
Potential pathologies responsible for UGIB	7.5	8.5	0.03
Medical management of variceal and non-variceal upper GI bleed	8	8	0.01
Understanding theoretical principles			
Adrenaline infiltration	7	8.5	<0.001
Clip placement	7	8	<0.001
Variceal banding	6.5	8	0.007
Thermal therapy	6	8.5	0.001
Hemospray use	5.5	8	<0.001
Sengstaken-Blakemore tube placement	5.5	7.5	0.02
Danis stent placement	5	8	0.04
Hands-on skills (For ST3 and above)			
Adrenaline infiltration	7	9	<0.001
Clip placement	6	9	0.001
Variceal banding	7	9	0.001
Thermal therapy	6	9	<0.001
Hemospray use	6	9	0.01
Sengstaken-Blakemore tube placement	5	8	0.002
Danis stent placement	4	9	0.001
Behaviour (only ST3 and above)			
Appropriate verbal instructions when applying haemostasis	5	9	<0.001
Other statistics			
Average endoscopy performed	583		
JAG-certified in endoscopy	10		

Assessing at an individual level, the course is most likely to benefit the gastroenterology trainees. However, even the consultants felt improvement in their managing capabilities, especially performing confidently during the procedures (Table 2).

**Table 2:** Subgroup Analysis Between Pre- and Post-Course’s Median Scores Based on Attendee’s (Based on Grade) Knowledge About UGIB, Knowledge of Procedure, and Demonstration of the Procedure and Behaviour A p-value of <0.05 was considered to be statistically significant. Scores were out of 10. N/A: Due to the low set of numbers, a p-value could not be calculated. UGIB, upper gastrointestinal bleeding

Attendee’s grade	Knowledge about UGIB			Knowledge of procedure			Demonstration of the procedure			Behaviour			Group p-value
	Pre-course median	Post-course median	p-Value	Pre-course median	Post-course median	p-Value	Pre-course median	Post-course median	p-Value	Pre-course median	Post-course median	p-Value	
Gastroenterology consultant	8	8	0.70	7.5	8	< 0.001	7.5	8	< 0.001	7	9.5	0.08	3.32
Gastroenterology registrars/fellows	8	9	0.002	6	9	9.78	5	9	1.62	4	9.5	0.01	5.55
Other registrars and consultants	7	9	0.002	5	9	0.006	5	9	0.001	5	9	N/A	1.90
Junior doctors	4	6	0.08	2	7	< 0.001	-	-	-	-	-	-	-
Nursing staff	5	7.5	<0.01	6	8	0.41	-	-	-	-	-	-	-

Gastroenterology consultants

A total of four gastroenterology consultants had attended the course. A mean of 2,375 endoscopic procedures were performed by the consultants. Three of the four consultants were JAG-certified for upper GI endoscopic procedures. In comparison of the pre-course and post-course’s median of scores given by the delegates, a statistically significant improvement was observed while comparing the knowledge (post-course median of 8; p < 0.001) and performing the aforementioned procedure (post-course median of 8; p < 0.001). However, no statistically significant improvement was observed when comparing the knowledge of UGIB or confidence in instructing while performing the procedure (pre-course vs. post-course). Overall, an improvement was observed, but it was not statistically significant.

Gastroenterology trainees/registrars

Eight gastroenterology trainees/registrars attended the course. The mean number of procedures undertaken by the registrars was 318.37. Five out of eight were JAG-certified for conducting upper GI endoscopic procedures. There was a vast improvement in the knowledge of the UGIB (post-course median of 9; p = 0.02) and the confidence/behaviour while instructing/performing the procedure (post-course median of 9.5; p = 0.01), which was statistically significant as well. Also, the knowledge of the various procedures improved drastically along with the ability to demonstrate the procedural skills of the registrars/fellows.

Registrars and consultants from other fields

One surgical consultant, one surgical registrar, and one acute medicine registrar had attended the course with a mean of 260 diagnostic endoscopic procedures performed. Two of them were JAG-certified. A statistical significance was observed while comparing the knowledge of UGIB (post-course median of 9; p = 0.002) and haemostasis procedures (post-course median of 9; p = 0.006) and in performing the procedure (post-course median of 9; p = 0.001) before and after the course.

Junior doctors

Three junior doctors/residents had attended the lectures on UGIB and procedures. The results also showed a statistically significant improvement in the knowledge of the procedures (post-course median of 7; p < 0.001).

Nursing staff

Nursing staff had also attended the lectures on UGIB and procedures. An overall improvement was observed, with statistically significant improvement in the knowledge of UGIB (post-course median of 7.5; p < 0.01).

**Figure 1:**
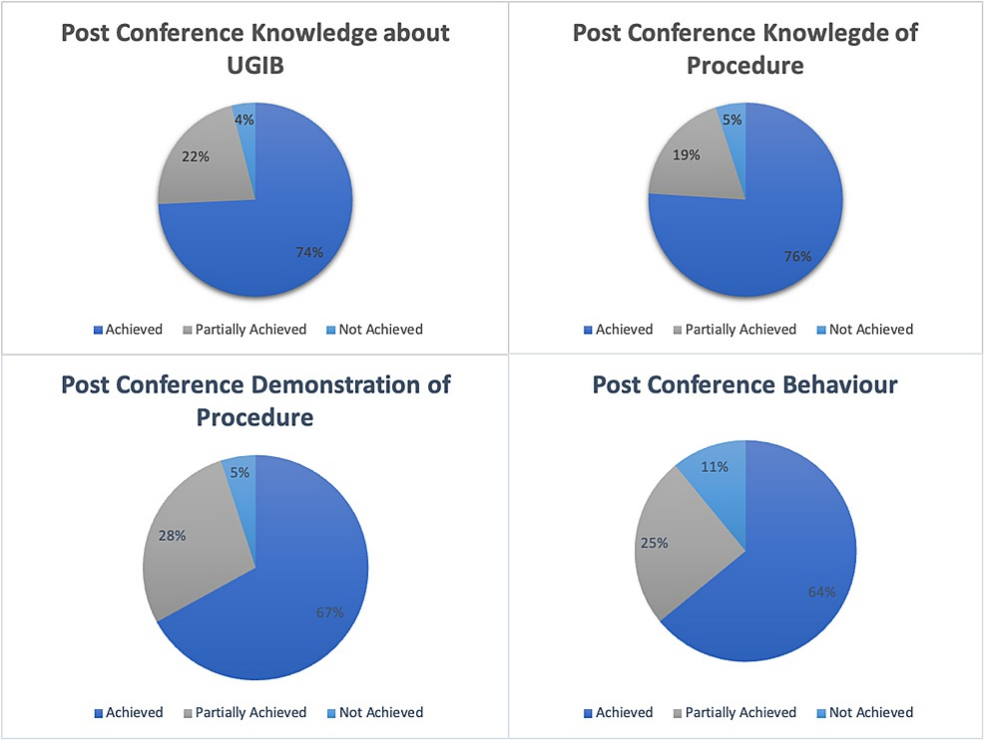
Post-Course Objective Achievements of Knowledge and Skills of the Delegates UGIB: upper gastrointestinal bleeding

Furthermore, the majority more than two-thirds) of the participants achieved their desired level of knowledge and procedural skills training post-course (Figure 1). The delegates were asked if their objectives were not achieved (did not understand/still cannot perform the procedures), partially achieved (understood but will need more knowledge or will be hesitant to perform the procedure or will need supervision), or fully achieved (understood and can perform the procedures independently).

## Discussion

AUGIB management is highly dependent on the quality of endoscopy performed in a timely manner, which itself is directly dependent on the number of haemostasis procedures performed previously. In our study, the mean endoscopic procedures performed by the delegates were 583. In a previous study, out of all endoscopies performed, only 5.8% were performed for haemostasis purposes, suggesting the lack of experience in haemostasis procedures in majority of the gastroenterologist due to limited exposure [19].

The present study in our knowledge is one of the first studies that suggests that conducting an AUGIB haemostasis course may help in standardising the training pattern of the gastroenterology trainees nationally. AUGIB is a well-known medical emergency that should be managed promptly to prevent any compromise in patient safety. Our study further shows that it benefits not only the gastroenterology trainees but also the gastroenterology consultants, trainees from other field, and nurses. This course is useful to many healthcare workers, but overall it significantly benefits the gastroenterology consultants and trainees. It could be applied to different staff members with slight modification in the course according to their roles and responsibilities in patient care. For example, paramedics and nurse practitioners may not need training in performing endoscopic interventions such as hands-on training on adrenaline injection, clip placement, variceal banding, thermal therapy, use of haemostatic powder (Hemospray), Sengstaken-Blakemore tube placement, and Danis stent placement.

It has been observed nationally that irrespective of grade, there is limited knowledge and confidence in managing AUGIB. A very recent study conducted by Segal et al. reported that only 31.5% (overall, specialty trainee [ST] in year 7 having approximately 60% while ST3 having 0%) of the trainees had the confidence to manage UGIB independently and further suggested that personal training if given to the trainees can help in boosting their confidence [12]. In our study, a statistically significant improvement in the knowledge and confidence of the gastroenterology trainees while instructing during the procedure/managing the UGIB was observed, which was not seen in the group of consultants (in which no statistically significant improvement was observed in confidence while instructing, which could be due to a large number of procedures performed already) (Table 2). However, it supports Segal et al.’s notion that training the registrar from the early stages will be the most beneficial outcome. For the ST7, this is pivotal as they will soon be put in the front line on the on-call rota for managing AUGIB.

The present course also helps in training the trainees with different methods of managing the UGIB. This will certainly be beneficial as previous data from 765 UK trainee portfolios reported that 50.7% had never placed a clip and 37.1% never performed band ligation [20], and the median number of haemostasis procedures performed up to completion of training was 42 [19]. Furthermore, from 2021, the training will be shortened from five years to four years [21]; hence, the number of opportunities shall decrease further. In addition to this, the general confidence and competencies of trainees will be reduced. Therefore, such courses are an accessible and key aid for trainees to get exposure to various forms of available options.

It is pertinent to notice that there is currently no minimum requirement of procedural numbers from either BSG or JAG to achieve competence in managing UGIBs. However, The European Section and Board of Gastroenterology and Hepatology (ESBGH) have recommended a bare minimum of 30 haemostatic procedures in order to achieve competence. A study conducted by Siau et al. [19] demonstrated that only 65% of the trainees were competent if we take ESBGH recommendations into consideration. The present course further becomes valuable as it allows the participants to perform procedures on the porcine models. Use of such models enables scenarios to be as realistic as possible but preventing any patient harm. Patient-centred care is imperative. The increase in practice and training of physicians results in better patient care. Ultimately, this is the course’s objective.

Recently, a similar JAG-approved haemostasis course was conducted, which also showed an improvement in the confidence of the delegates attending it (mostly attended by the gastroenterology consultants or JAG-certified consultants) [11]. Though our study did not show a statistically significant improvement in confidence, it helped in advancing the knowledge and procedural skills of the gastroenterology consultants (Table 2) and further condoning the fact that haemostasis course will be a big boost in managing AUGIB. At present, a national training course “Train the Bleed Trainers” is being developed, which may become a part of JAG certification in future, thus supporting the importance of our haemostasis course [22].

Our study also shows the importance of conducting this course for consultants and registrars of other branches and junior doctors who may be interested in selecting gastroenterology as their future specialty. There was a significant improvement observed in consultant and registrars of other branches in UGIB knowledge (median of 9; p = 0.002), knowledge regarding endoscopic procedure with regards to haemostasis (median of 9; p = 0.006), and performing them (median of 9; p = 0.001) post-course. Even junior doctors had an improvement in their procedural knowledge (median of 7; p < 0.001) post-course (Table 2). Nurses too had an improvement in their knowledge regarding UGIB post-course. It is paramount that staff members with patient contact have a certain degree of understanding of the treatment of a UGIB and know when to alert seniors. The prognosis for patients is improved with time-efficient management. If more staff members are familiar with this presentation, it will allow for quicker diagnosis. Even a slight improvement in knowledge regarding UGIB will expedite treatment.

Including this haemostasis course in gastroenterology training curriculum will not only modernise the training by keeping trainees up to date with their skill set but will also improve the training quality in managing AUGIB of the gastroenterology trainees and boost confidence, knowledge, and skills of delegates attending it. We believe that this course can be used nationally in order to overall improve training and positively contribute to patient care and safety.

Limitations

The present study was conducted on a smaller number of participants. Hence, we have used a non-parametric test (Wilcoxon signed-rank test) to compare the results. Furthermore, this study involves limited representation from gastroenterology consultants and registrars. We were also unable to stratify analysis by subspecialty, length of training, or any previous gastroenterology experience globally. Furthermore, as the study was based on self-assessed questionnaire, there is a possibility of a self-assessment bias. A large-scale study would be beneficial in confirming the findings.

## Conclusions

The knowledge, skills, and confidence in managing UGIB independently of the delegates improved significantly with this one-day haemostasis course. Conducting such courses at regular intervals for all medical and nursing staff, especially the gastroenterology trainees, will help improve the management of UGIB and as a result may help reduce the mortality and morbidity nationally. We hence recommend to include this course in GI training on yearly basis.
